# Age- and sex-related difference of lipid profile in patients with ischemic stroke in China

**DOI:** 10.1097/MD.0000000000010930

**Published:** 2018-06-18

**Authors:** Pingsen Zhao, Sudong Liu, Zhixiong Zhong, Jing Liu

**Affiliations:** a Clinical Core Laboratory, Meizhou People's Hospital (Huangtang Hospital); b Center for Precision Medicine, Meizhou People's Hospital (Huangtang Hospital), Meizhou Hospital Affiliated to Sun Yat-sen University; c Guangdong Provincial Engineering and Technology Research Center for Molecular Diagnostics of Cardiovascular Diseases; d Meizhou Municipal Engineering and Technology Research Center for Molecular Diagnostics of Cardiovascular Diseases.; e Meizhou Municipal Engineering and Technology Research Center for Molecular Diagnostics of Major Genetic Disorders; f Center for Cardiovascular Diseases, Meizhou People's Hospital (Huangtang Hospital), Meizhou Hospital Affiliated to Sun Yat-sen University, Meizhou, P. R. China.

**Keywords:** cholesterol, dyslipidemia, ischemic stroke, lipid metabolism

## Abstract

Age- and sex-related differences of lipid profiles were not well understood among ischemic stroke patients in China. Our study aimed to investigate the relationship between lipid abnormalities and ischemic stroke in China. A retrospective analysis was performed involving 2074 patients with ischemic stroke who admitted to Meizhou People's Hospital (Huangtang Hospital), Meizhou Hospital Affiliated to Sun Yat-sen University between January 1, 2014 and March 1, 2017. Our study indicated that there were significant differences in total cholesterol (TC), high-density lipoprotein cholesterol (HDL-C), non-high density lipoprotein cholesterol (non-HDL), and Apolipoprotein A (ApoA) between male and female patients. Meanwhile, different lipid levels including TC, TG, and ApoA were observed for patients of various age groups. The nonelderly patients tended to have higher lipid levels than elderly patients. Isolated low HDL (namely, normal LDL + low HDL + normal TG) was the most common lipid abnormalities in patients. In conclusion, there was age- and sex-related difference in lipid profiles among patients with ischemic stroke. Age, sex as well as some other factors should be carefully considered for lipid management in patients with ischemic stroke in China.

## 1 Introduction

Stroke is ranked as the second most common single cause of death and is a major global cause of long-term disability with up to 1/6 survivors remaining permanently disabled.^[1,2]^ In China, it was reported that mortality of stroke increased stably in the last 10 years and in 2014 alone, 837.3 thousand and 1023.4 thousand persons died from stroke in rural and urban areas, respectively.^[3]^ Stroke has become a major threat to public health and the situation is likely to worsen with the aging of population and unhealthy lifestyle.^[4]^

Stroke is generally divided into ischemic stroke (55%–90%) and hemorrhagic stroke (12%–35%). Caused by cerebral ischemia, ischemic stroke is the major subtype that leads to neurological dysfunction.^[5,6]^ Dyslipidemia, which is defined as the presence of abnormal levels of lipid in the blood including elevated total cholesterol (TC), elevated low-density lipoprotein cholesterol (LDL-C), elevated triglycerides (TG), and reduced-high density lipoprotein cholesterol (HDL-C), is a well-known risk factor for cardiovascular and cerebrovascular disease (CVD).^[7]^ Although large-scale evidences from randomized trials have proven that statin therapy reduced the risk of major vascular events by lowering low-density lipoprotein (LDL) levels,^[8]^ epidemiologic association between cholesterol levels and stroke remains debatable.^[9]^ Grobbee et al^[10]^ found that each 1-mmol/L increase in TC level enhanced the risk of ischemic stroke by about one-quarter. Similar evidences showed that the risk for ischemic stroke increased as TC level exceeded 200 mg/dL, even to the extent of more than double when exceeded 280 mg/dL.^[11,12]^ However, other researchers failed to find such link.^[13,14]^ Some studies suggested that dyslipidemia raised risk for stroke, but such relationship was often confounded by the types of stroke.^[15]^

Stroke incidence also differed between male and female population. Although the incidence of stroke was greater among men, women suffer worse outcomes from the disease.^[16]^ Until now, it is unclear the exact reason for this phenomenon, but several factors such as dyslipidemia, hypertension (HTN), diabetes mellitus (DM), cigarette smoking, and alcohol drinking has been studied to explain the sex-related difference in stroke outcome. Some studies have reported a correlation between stroke and menopause, which caused changes in arterial structure and other biochemical factors and may contribute to atherosclerosis.^[17,18]^ Age was suggested to be a crucial risk factor for stroke. Reports have suggested that about 80% strokes occurred in individuals aged >65 years, among which 50% were aged ≥70 years and nearly 25% aged >85 years.^[4]^

Age- and sex-related dyslipidemia has been previously reported in patients with acute myocardial infarction (AMI) in east China.^[19]^ To our knowledge, the pattern of dyslipidemia among patients with ischemic stroke in China remains unclear. Identification of specific risk factors may help to further improve outcomes following stroke, and such information may even help to devise practical preventive and interventional approaches to stroke care in these subpopulations.

In the present study, we examined the lipid profiles of a large scale of patients with acute ischemic stroke at admission from January 1, 2014 and March 1, 2017 in Meizhou People's Hospital (Huangtang Hospital), Meizhou Hospital Affiliated to Sun Yat-sen University. We evaluated the age- and sex difference of lipid levels, as well as other risk factors like smoking, drinking, hypertension, and diabetes in patients with acute ischemic stroke.

## 2 Methods

### 2.1 Subjects and study procedures

A total of 2074 acute ischemic stroke admissions between January 1, 2014 and March 1, 2017 were included in this study. Patients were diagnosed with ischemic stroke as meeting the guidelines for management of ischemic stroke and transient ischemic attack 2008.^[20]^ The presence of new lesions was confirmed by Magnetic Resonance Imaging (MRI) scan and acute infarction that occurred within 2 weeks was included for analysis. Exclusion criteria included cerebral hemorrhage, sequelae of cerebral infarction, neurological headache, concomitant complications of severe anemia or severe heart diseases, tumors or infection. Besides, patients currently receiving lipid-lowering treatment or drugs were also excluded. This study was approved by Human Ethics Committees of Meizhou People's Hospital (Huangtang Hospital), Meizhou Hospital Affiliated to Sun Yat-sen University, Guangdong province, China. All the patients have signed the informed consent.

### 2.2 Lipid profile testing

The fasting lipid profiles were examined the next morning after admission by a Cobas 6000 analyzer series and kits (Roche Diagnostics, Basel, Switzerland). Additionally, 4 nontraditional lipid profiles (nonHDL, TC/HDL, LDL/HDL, TG/HDL) were also analyzed in this study. Non-HDL was defined as TC minus HDL (non-HDL = TC – HDL). TC/HDL, LDL/HDL, TG/HDL represented the ratio of TC to HDL, LDL to HDL, and TG to HDL, respectively.

The following factors were recorded: sex, age, hypertension, diabetes mellitus, and history of smoking and drinking (smoking or alcohol drinking within a year before admission).

### 2.3 Data analysis

All statistical analyses were performed using GraphPad Prism 5.5 software. Continuous data are presented as mean ± standard deviation (SD). Unpaired *t* test was used to compare between 2 groups, whereas analysis of variance was used for comparison among 3 or more groups. Discrete variables, expressed as counts and percentages, were analyzed by *χ*^*2*^-square or Fisher exact test. *P* < .05 was considered statistically significant.

## 3 Results

### 3.1 Clinical characteristics of enrolled subjects

Totally, 2074 ischemic stroke patients (1386 men and 688 women) were eligible and included in our analysis. The patients aged 17 to 97 years, including 1111 nonelderly (<65 years) and 963 elderly (≥ 65 years).^[21]^ Clinical characteristics of the enrolled subjects were presented in Table 1. Compared with the nonelderly, the elderly tended to have lower levels of TC, TG, LDL, ApoA, and ApoB, as well as the 4 nontraditional lipid profiles. Meanwhile, females seemed to have higher levels of TC, HDL, ApoA, ApoB, and nonHDL than males in both nonelderly and elderly. Higher level of LDL was solely observed in elderly females than males, whereas higher level of TC/HDL, LDL/HDL, and TG/HDL were solely observed in nonelderly males than females.

**Table 1:**
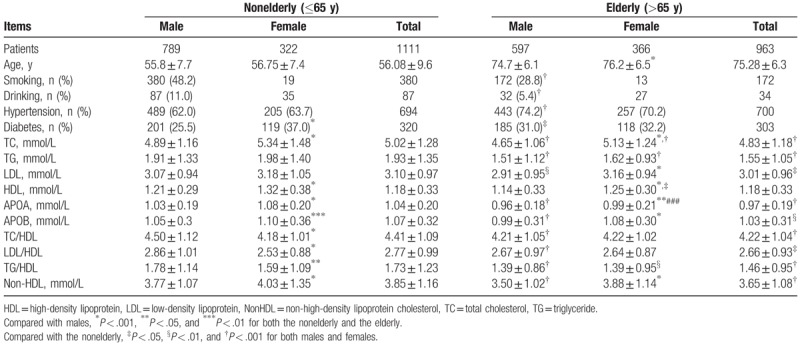
Clinical characteristics for patients with ischemic stroke.

### 3.2 Age- and sex-related difference in TC, TG, HDL, and LDL for patients with ischemic stroke

**Figure 1:**
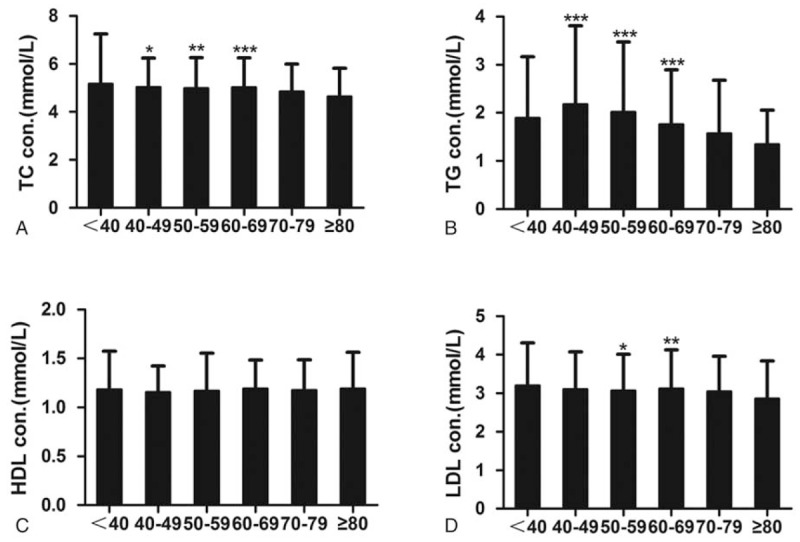
Lipid profiles of patients with ischemic stroke in various age groups (N = 2074). (A) TC level in ischemic stroke patients of various age groups; (B) TG levels in ischemic stroke patients of various age groups; (C) HDL levels in ischemic stroke patients of various age groups; (D) HDL levels in ischemic stroke patients of various age groups. Differences between groups were compared using one-way analysis of variance with Tukey post test. ^∗^*P* < .05, ^∗∗^*P* < .01, and ^∗∗∗^*P* < .001. HDL = high-density lipoprotein, LDL = low-density lipoprotein, TC = total cholesterol, TG = triglyceride.

**Figure 2:**
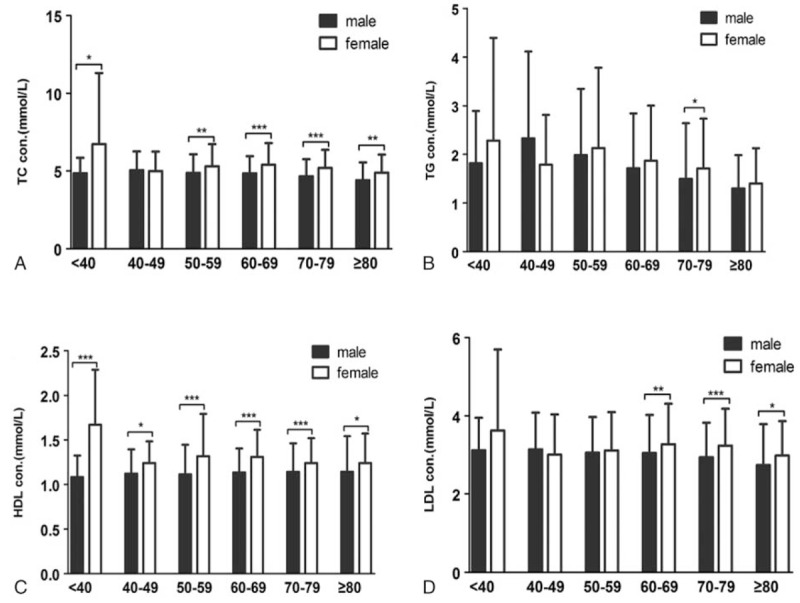
Sex-related difference of lipid profile in patients with ischemic stroke in various age groups (N = 2074). (A) Comparison of TC in ischemic stroke patients between male and female; (B) comparison of TG in ischemic stroke patients between male and female; (C) comparison of HDL in ischemic stroke patients between male and female; (D) comparison of LDL in ischemic stroke patients between male and female. Data were compared using student t test. ^∗^*P* < .05, ^∗∗^*P* < .01, and ^∗∗∗^*P* < .001. HDL = high-density lipoprotein, LDL = low-density lipoprotein, TC = total cholesterol, TG = triglyceride.

In our analysis, patients were divided into 6 age groups. Age-related lipid profiles were shown in Figure 1. Significant difference could be observed in TC, TG, and LDL among various age groups (*P* < .01). TC and TG levels in 40 to 49, 50 to 59, and 60 to 69 years age groups were significantly higher than those in ≥80 years age group. LDL level in 50 to 59 and 60 to 69 years age groups were significantly higher than that in ≥80 years age group. Figure 2 showed the sex-related lipid profiles. Surprisingly, female patients have significant higher HDL in all the 6 age groups. Female patients also have higher levels of TC in all age groups except 40 to 49. Sex-related difference was also seemed in LDL in age group 60 to 69, 70 to 79, and ≥80. Little difference was seen in TG in various age groups except in 70 to 79, which was in accordance with data shown in Table 1.

### 3.3 Comparisons of lipid abnormality between elderly and nonelderly patients with ischemic stroke

According to the Guideline for the Prevention and Treatment of Chinese Adult Dyslipidemia 2007,^[22]^ lipid of either LDL ≥3.37 mmol/L (130 mg/dL), HDL <1.04 mmol/L (40 mg/dL), TG ≥1.70 mmol/L (150 mg/dL) or TC ≥5.18 mmol/L (200 mg/dL) was defined as lipid abnormality. Thus, there are 4 kinds of single lipid abnormality and 7 possible kinds of combined lipid abnormality. Table 2 showed the single lipid abnormality in patients with ischemic stroke. It is indicated that male patients are more prevalent in low HDL than female patients (40.4% vs. 22.2%, *P* < .05). The incidence of high LDL and high TC in female patients were significantly higher than those in male patients (38.1% vs. 32.0%, *P* < .05 and 47.8% vs. 35.5%, *P* < .05, respectively). Nonelderly males seemed to have higher incidence rate of high LDL, high TG, and high TC than elderly males, and nonelderly females seemed to have lower incidence rate of low HDL and higher incidence rate of TG than elderly females.

**Table 2:**
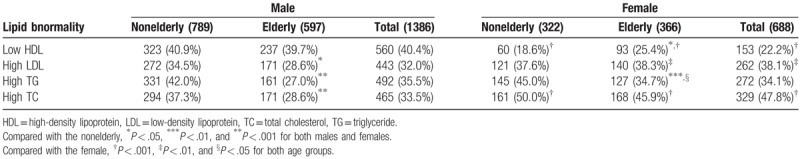
Single lipid abnormality in patients with ischemic stroke.

The Table 3 showed the distribution of 7 types of combined dyslipidemia in ischemic stroke patients. It was shown that dyslipidemia occurred in >70% of ischemic stroke patients and single dyslipidemia was more prevalent than mixed dyslipidemia (41.5% vs. 29.4%, *P* < .05). Single low HDL (namely, low HDL + normal LDL + normal TG) and single high LDL (namely, high LDL + normal HDL + normal TG) were the most prevalent types of dyslipidemia for ischemic stroke, with an incidence rate of 15.7% and 14.1%, respectively.

**Table 3:**
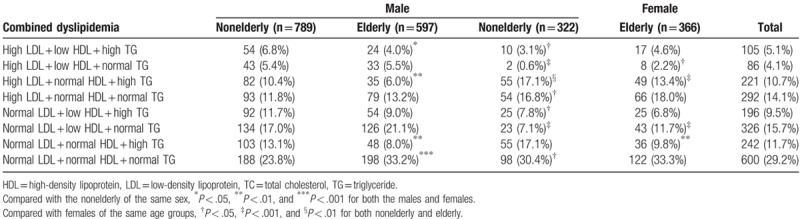
Combined dyslipidemia in patients with ischemic stroke.

### 3.4 Age- and sex-related difference of lipid abnormality

**Figure 3:**
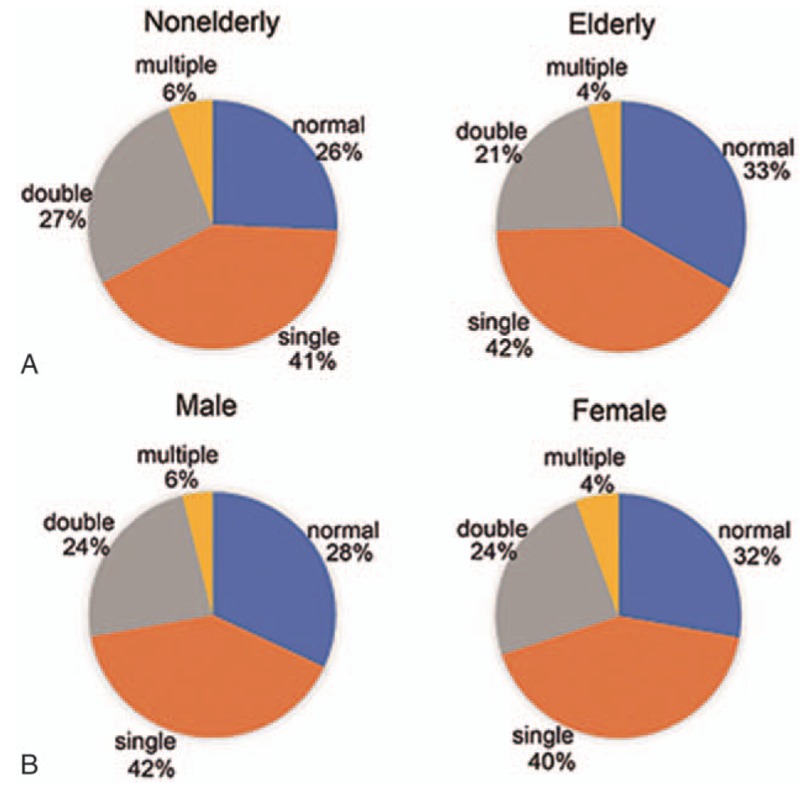
Single-, double- and multiple lipid abnormality distribution in patients with ischemic stroke. (A) Comparison of single-, double-, and multiple lipid abnormality between nonelderly and elderly; (B) comparison of single-, double-, and multiple lipid abnormality between male and female.

Based on the abnormality of lipid profile, patients were divided into 4 groups including “normal,” “single,” “double,” and “multiple.” As shown in Figure 3A, lipid abnormality was more prevalent in nonelderly patients (74% vs. 67%, *P* < .05). The proportion of double- abnormality in nonelderly patients was significantly higher than that in elderly patients (27% vs. 21%, *P* < .05), whereas no difference was seen in single- and multiple groups. Figure 3B showed the sex-related combined dyslipidemia, the proportion of single- and multiple abnormalities in males were slightly higher than that in females, although the difference was not statically significant. Multivariate logistic regression analysis was applied to establish the risk factors in patients with ischemic stroke (Table 4). It was shown that sex was a significant risk factor for lipid abnormality in patients with ischemic stroke, whereas age was a slight protective factor.

**Table 4:**
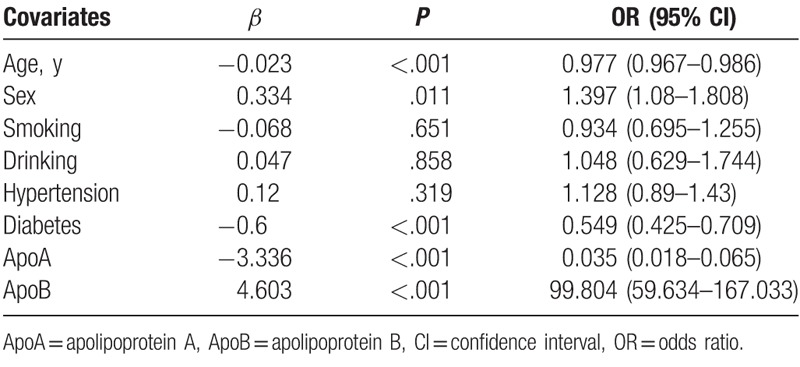
Multivariate logistic regression analysis model for dyslipidemia in patients with ischemic stroke.

## 4 Discussion

In the present study, we investigated the lipid profile of >2000 hospitalized ischemic stroke patients. Our data showed that: there was significant age- and sex difference in lipid profiles; nonelderly patients had more prevalent and severe lipid abnormality; female stroke patients seemed to have higher levels of TC, TG, and LDL; single low HDL and single high LDL were the most common lipid abnormality.

Until now the relationship between cholesterol levels and stroke has been extensively studied and increasing evidences suggested that dyslipidemia was an important risk factor for stroke.^[23]^ Glasser et al led a follow-up study on association of lipid levels and incident stroke, and found that LDL and non-HDL baseline levels were significantly linked to the risk of ischemic stroke.^[9]^ Bowman et al^[24]^ found that mortality of stroke was positively related to TC level in young women and inversely related to in subjects of 60 to 70 years. In a recent study, Xu et al^[25]^ found that dyslipidemia was associated with recurrent stroke risk. These findings highlighted the importance of understanding the characteristics of dyslipidemia in patients with stroke. Our study included a large scale of ischemic stroke cases and analyzed the dyslipidemia in stroke. Our data suggested that dyslipidemia was very common in patients with ischemic stroke. We also found that age and sex were risk factors affecting lipid levels, and dyslipidemia patterns were different between nonelderly and elderly, as well as male and female patients.

Hypercholesterolemia promoted atherosclerosis because increased oxidized LDL cholesterol resulted in endothelial dysfunction.^[26]^ Meta-analysis found a 50% increased risk of ischemic stroke among those in the highest quintile of fasting TG compared with those in the lowest quintile.^[13,27]^ Also, a population-based cohort study composed of approximate 14,000 subjects found that elevated nonfasting TG enhanced the risk to develop ischemic stroke in both men and women.^[28]^ It was increasingly recognized that there were differences between male and female in relation to stroke. Evidence indicated that compared with male stroke patients, female stroke patients tended to have worse outcomes, for example, more severe disability.^[16]^ In our study, female stroke patients had higher proportion of dyslipidemia, especially the in TC and LDL levels.

Epidemical studies showed that over 80% strokes occurred in the elderly (≥65 years) and this could be explained by the presence of greater risk factors that increased with age, such as atrial fibrillation and hypertension.^[4]^ It was reported that TC level in women increased with age, whereas in men tended to remain stable.^[16]^ However, in our study, cholesterol levels including TC, TG, and LDL were significantly higher in nonelderly than in elderly. These data indicated that although stroke was traditionally considered as a disease of the elderly, more attention should be paid to stroke risk factor recognition and management in younger population.

Diet has been proved to be a very important factor that influenced lipid level.^[29]^ The red wine contained rich resveratrol and polyphenol contents, which counteracted hypercholesterolemia.^[30]^ Fish oil supplementation had always been considered to improve lipid profile.^[31]^ Soy milk and its derivatives in the common diet could significantly reduce plasma levels of all lipids (TC, TG, and LDL).^[32]^ Meanwhile, many studies have already suggested that plant sterols actively influenced lipid profile as mean LDL reduction after consumption of plant sterol-supplemented foods ranges from 5.9% to 10.4%.^[33]^ City Meizhou is the home of hakka population, and there are plenty of traditional foods with high-fat in this region. Local people are accustomed to cook with pork oil and eat fried snacks daily. These diet habits would contribute to the lipid levels of subjects included in this study. However, as this was a retrospective study, we were unable to obtain the diet information of patients because it was not on the medical record.

### 4.1 Limitation

There are some limitations for this study. First, we did not have healthy control group in this study. We tried to include healthy population who came to hospital to have body examination, but most healthy volunteers did not test for lipid level. So the present study aimed at investigating the dyslipidemia characters in patients with stroke rather than prove the association between dyslipidemia and stroke. Second, the retrospective nature of this study made some important information absent, such as diet and education background.

## 5 Conclusion

This study demonstrated the age- and sex-related difference in lipid profiles among ischemic stroke patients from China. More prevalent and more severe dyslipidemia was observed in the nonelderly than elderly, and women seemed to have higher proportion of lipid abnormality than men. Single low HDL and single high LDL were the most common lipid abnormality. These findings would be instructive for lipid management and reduce dyslipidemia in stroke patients.

## Acknowledgments

The author thank other colleagues whom were not listed in the authorship of Department of Neurology, Center for Cardiovascular Diseases, Clinical Core Laboratory and Center for Precision Medicine, Meizhou People's Hospital, Meizhou Hospital Affiliated to Sun Yat-sen University for their helpful comments on the manuscript.

## Author contributions

**Conceptualization:** Pingsen Zhao.

**Data curation:** Pingsen Zhao, Zhixiong Zhong.

**Formal analysis:** Pingsen Zhao, Sudong Liu, Jing Liu.

**Funding acquisition:** Pingsen Zhao, Zhixiong Zhong.

**Investigation:** Pingsen Zhao, Sudong Liu, Zhixiong Zhong, Jing Liu.

**Methodology:** Pingsen Zhao, Sudong Liu, Zhixiong Zhong, Jing Liu.

**Project administration:** Pingsen Zhao.

**Resources:** Pingsen Zhao.

**Software:** Pingsen Zhao, Jing Liu.

**Supervision:** Pingsen Zhao.

**Validation:** Pingsen Zhao.

**Writing – original draft:** Pingsen Zhao, Sudong Liu, Zhixiong Zhong.

**Writing – review & editing:** Pingsen Zhao.
